# Fostering Sustainable Biomedical Research Training in Mozambique: A Spin-Off of the Medical Education Partnership Initiative

**DOI:** 10.5334/aogh.3684

**Published:** 2022-08-02

**Authors:** Emília Virgínia Noormahomed, Sérgio Noormahomed, Delfina Hlashwayo, Emília Martins, Mamudo Ismail, Stephen W. Bickler, Jean Nachega, Manuel Mahoche, Kim E. Barrett, Constance A. Benson, Robert T. Schooley

**Affiliations:** 1 Department of Microbiology, Faculty of Medicine, Universidade Eduardo Mondlane (UEM), Maputo, MZ; 2 Department of Medicine, Division of Infectious Diseases, University of California, San Diego (UCSD), US; 3 Mozambique Institute for Health Education and Research (MIHER), Maputo, MZ; 4 Department of Biological Sciences, Faculty of Sciences, Universidade Eduardo Mondlane (UEM), Maputo, MZ; 5 Department of Pathology, Faculty of Medicine, Universidade Eduardo Mondlane (UEM), Maputo, MZ; 6 Division of Pediatric Surgery, Rady Children’s Hospital, UCSD, USA; 7 Department of Medicine, Division of Infectious Diseases, Stellenbosch University Faculty of Medicine and Health Sciences, Cape Town, South Africa; 8 Department of Epidemiology, Infectious Diseases and Microbiology, and Center for Global Health, University of Pittsburgh, Pittsburgh, PA, USA; 9 Department of Epidemiology, Johns Hopkins Bloomberg School of Public Health, Baltimore, MD, USA; 10 Department of International Health, Johns Hopkins University, Bloomberg School of Public Health, Baltimore, MD, USA; 11 Faculty of Health Sciences, Lúrio University, Nampula, MZ; 12 Division of Gastroenterology, Department of Medicine, University of California San Diego (UCSD), USA; 13 Department of Physiology and Membrane Biology, University of California Davis School of Medicine, USA; 14 Department of Medicine Division of Infectious Diseases and Global Public Health, University of California, San Diego, California, USA

**Keywords:** research capacity building, faculty development, early career research development, mentorship, MEPI, Mozambique

## Abstract

**Background:**

The further development of research capacity in low- and middle-income countries is critical to the delivery of evidence-based healthcare, the design of sound health policy and effective resource allocation. Research capacity is also critical for the retention of highly skilled faculty and staff and for institutional internationalization.

**Objectives:**

We summarize the accomplishments, challenges and legacy of a five-year program to train biomedical researchers entitled “Enhanced Advanced Biomedical Research Training for Mozambique (EABRTM)”.

**Methods:**

A program conducted from 2015–2021 built upon the Medical Education Partnership Initiative to develop research capacity at Eduardo Mondlane University (UEM) and allied institutions. The project included design and implementation of postgraduate training programs and bolstered physical and human research infrastructure.

**Findings:**

The program supported development and implementation of UEM’s first doctoral (Bioscience and Public Health) and master (Biosciences) programs with 31 and 23 students enrolled to date, respectively. Three master programs were established at Lúrio University from which 176/202 (87.1%) and 107/202 (53.0%) students obtained a Postgraduate Diploma or master’s degree, respectively. Scholarships were awarded to 39 biomedical researchers; 13 completed master degrees, one completed a PhD and five remain in doctoral studies. Thirteen administrative staff and four biomedical researchers were trained in research administration and in biostatistics, respectively. A total of 119 courses and seminars benefited 2,142 participants. Thirty-five manuscripts have been published to date in peer-reviewed international journals of which 77% are first-authored by Mozambicans and 44% last-authored by Africans. Sustainability was achieved through 59 research projects awarded by international agencies, totaling $16,363,656.42 and funds ($ 7,319,366.11) secured through 2025.

**Conclusions:**

The EABRTM program substantially increased research and mentorship capacity and trained a new generation of biostatisticians and research administrators. These programmatic outcomes significantly increased the confidence of early stage Mozambican researchers in their ability to successfully pursue their career goals.

## Background

Mozambique’s population of 30,06 million includes 62.9% living below the income poverty line and 66% in rural areas. Life expectancy (61 years) is below the African average and the adult literacy rate is 60.7% [1,2,3,4,5]. Mozambique has one of Africa’s lowest per capita rates of higher education at both the undergraduate and the postgraduate levels [3]. From 2004–2010, only 22% and 11% of Mozambican university faculty had master’s degrees or PhD degrees, respectively. Most advanced degrees were obtained abroad. Only 4.5% (4,550) of Mozambique’s university students were enrolled in master’s or doctoral programs [3].

Mozambique also has among the fewest health professionals per capita (0.4 physicians and 4.12 nurses and midwives per 10,000 people), despite a national disease profile that is dominated by infectious diseases such as HIV/AIDS (13%), malaria (40%), diarrheal and respiratory infections (29%), tuberculosis (361/100,000 inhabitants) and neglected tropical diseases such as intestinal parasites (53%), lymphatic filariasis (13%), and schistosomiasis (47%). There are also substantial rates of chronic malnutrition (43%) and mortality from non-communicable diseases (28%) [2,3,6,7,8].

After gaining independence in 1975, a 16-year-long civil war challenged Mozambique’s economy and slowed development of its only public university (the Lourenço Marques University, now the Eduardo Mondlane University (UEM)) [2,9]. UEM eventually regained its footing and student enrollment reached 39,078 by 2015 [2,10]. In 1999, UEM launched the first of several master’s and PhD programs in areas critical to national development. These programs relied heavily on external support from bilateral cooperation and NGOs to provide faculty and to train UEM faculty members, the latter mostly with master’s and PhD degrees obtained abroad [2,3,11]. By 2015, the UEM Faculty of Medicine had 187 university lecturers. Of those, only 19 (10%) and 34 (18%) held master’s and PhD degrees, respectively. Of UEM’s 50-member veterinary faculty, 8 (16%) held licentiate degrees; 25 (50%) and 17 (34%) held master’s and PhD degrees respectively. The majority of the 274 lecturers in UEM’s Faculty of Sciences had post graduate degrees: 115 (42%) with master’s and 87 (32%) with doctorates. Publications were sparse and most resulted from research conducted abroad in fulfilment of the requirements for postgraduate degrees [11,12]. Upon returning to Mozambique, ongoing research engagement was challenged by limited financial resources, minimal dedicated research time, a poor research infrastructure with few mentors, poor access to the scientific literature, and limited English proficiency. Returning faculty interested in research pursuits faced limited career progression pathways [1,3,12,13,14,15]. By December 2009, less than 10% of African medical schools were involved in sponsored research [11,12,15,16,17]. Mozambique was not among the top 10 countries from WHO African region publishing in peer-reviewed journals [3,18,19].

The Instituto Nacional de Saúde (INS) under the Ministry of Health (established in 2004), the Manhiça Health Research Centre (*Centro de Investigação em Saúde de Manhiça* – CISM), the main UEM teaching hospital (the Maputo Central Hospital (MCH)) and a handful of non-governmental organizations conduct biomedical research in Mozambique and topics of investigation are generally dictated by donor priorities [1,14,15,20].

In 1985, the Mozambican Government created the Universidade Pedagógica de Mozambique (UPM), initially to train professors for the education system. Later, in 2006 and 2009, in the north and central part of Mozambique (located in Nampula and Tete province), the most populated but underserved regions in the country, two more universities were formed to train physicians and other professionals in Health Sciences: the Lúrio University (UniLúrio) and the Zambeze University (UniZambeze) [12].

In 2010, an existing collaboration between UEM and the University of California San Diego (UCSD) was leveraged by the Medical Education Partnership Initiative (MEPI), funded by the US Department of State under the President’s Emergency plan for AIDS Relief (PEPFAR) and the US National Institutes of Health (NIH) to accelerate research infrastructure development [2,21,22]. As an outgrowth of MEPI, we designed and implemented the Enhanced Advanced Biomedical Research Training for Mozambique (EABRTM) program (also funded by NIH) to train a new generation of biomedical researchers, and to enhance administrative research infrastructure at UEM and allied Mozambican research institutions.

This manuscript summarizes the impact of the EABRTM program and reflects on how the program contributed to a strengthening of biomedical research in Mozambique and north-south and south-south collaboration, and enumerates the challenges, lessons learned and the steps forward.

## The Case Study/Methods

This is a longitudinal case study conducted between 2015–2022. The data sources were annual progress reports, minutes from meetings and proceedings from international and national conference calls.

The trainees were recruited amongst faculty members and researchers from UEM, UniLúrio, INS, MCH and the Ministry of Health.

### Program background

As outlined above, the EABRTM sought to build upon MEPI’s goals and achievements to train a new generation of leaders in biomedical research in Mozambique.

Under MEPI, guided by a needs assessment led by a team of senior faculty from UEM, UCSD, and other participating Brazilian and Portuguese universities, we identified obstacles hampering Mozambique’s biomedical research productivity. Our initial steps were to implement master’s programs at UniLúrio, and to develop short courses in research methods, manuscript and grant writing, submission and management. The curriculum included bioethics, good clinical practices, biostatistics, Responsible Conduct of Research (RCR), and bioinformatics. Wet laboratories were enhanced and UEM laboratory staff received advanced training. A mentorship system was established [3,12,23]. We also created a Research Support Center, the Mozambique Institute for Health Education and Research (MIHER), to provide administrative and grant management to the scientific community at UEM and allied institutions. We identified funding agencies interested in supporting training and research activities in Mozambique [12]. The program established a Training Advisory Committee (TAC) that included senior faculty members from UEM, UCSD and Stellenbosch University dedicated towards the training and mentorship of biomedical researchers.

### Main Targets of the Program

We defined 6 main targets and outcomes as follows:

1. *Designing new Postgraduate programs and supporting the implementation of existing ones*

At UniLúrio, we supported the consolidation and expansion of three master’s programs (Tropical Medicine and Global Health (MTMGH), Health Professional Education (MHPE), and Nutrition and Food Security (MNFS)) that were initially developed with MEPI support [3]. At UEM, in 2021, we also designed and supported the initiation of the master’s of Biosciences program with three tracks: Parasitology, Microbiology and Neurosciences, and in 2017, we supported the implementation of the doctoral program in Biosciences and Public Health (DPBPH) with support from another NIH D43 TW010568 grant.

Each master’s program included a first year of didactic content (preparatory modules (15 credits), basic modules (45 credits) and advanced modules (60 credits)), a second year of seminar discussions on potential dissertations, and the research itself, culminating in a final examination. The general structure of our masters’ programs specified two years of activity with a total of 120 credits according to the National Credit Transfer and Accumulation System, in which each academic credit corresponds to 30 hours of interaction with the lecturer/mentor/supervisor [3].

Upon completion of preparatory and basic modules, a postgraduate diploma is awarded. This is followed by the advanced modules in the second year, which include seminars to elucidate dissertation topics under at least one mid-experienced supervisor. On completion of the research project, the student undergoes an examination by a jury of three scientists in the area; those passing the examination are awarded a master’s degree [3].

For the postgraduate programs, we enrolled candidates wishing to pursue postgraduate degrees, regardless of whether they were working in public, private or nongovernmental institutions. These candidates were selected according to the criteria defined by the Ministry of Higher Education for pursuing postgraduate studies. Those holding faculty positions and researchers at participating institutions were given priority for selection [3].

2. *Development and implementation of short courses (core modules) on research methods and didactic topics*

Senior mentors developed short courses (courses, seminars and workshops) on research methods, some of them developed under MEPI [12] for participating postgraduate students, junior mentors and research administrators.

3. *Training of biomedical researchers (faculty members and researchers at master’s and PhD degrees) and establishment of a mentorship system*

Biomedical researchers receiving advanced training included graduate and postgraduate faculty members, early career researchers and physicians working concurrently as research staff or faculty in participating institutions. The program was initially designed for ten junior mentors interested in strengthening their research and mentorship skills and willing to mentor other emerging researchers. Eligible candidates submitted applications outlining proposed research projects. Those selected by a dedicated jury conducted mentored research and participated in relevant short courses. During years 2 to 4, we planned to recruit 8 faculty members or researchers to enroll in the master’s or PhD programs. Scholarship support included stipends, payment of tuition and fees, funding for research work and continuous mentoring.

4. *Research administration development via training of research administrators*

Junior and senior research administrators were trained in Mozambique and at UCSD through courses on grants management and administration, and through participation in the annual conferences of the Society of Research Administrators and related workshops. These courses included fundamentals of research administration, development and submission of research proposals, administration and monitoring of grant awards, compliance with sponsor requirements, and maintenance of electronic platforms.

5. *Development of a Biostatistics Unit to support biomedical researchers*

This was done by further training of our trainees in biostatistics under the supervision of two midcareer faculty members.

6. *Fostering North-South and South-South collaboration*

These efforts included joint manuscript and grant writing, and networking meetings at international scientific meetings, including the African Forum for Research and Education in Health (AFREhealth), the D43 MEPI junior faculty meetings and the Consortium of Universities for Global Health (CUGH).

### Program evaluation

Program evaluation at an *individual level* was based on accomplishments of trainees provided with either full or partial scholarships. These included the number of conference presentations and manuscripts published. Trainee publications were confirmed by PubMed author field searches. The baseline scientific output of our trainees (defined as the number of publications attributed to them before their participation in the program) was compared to the number of publications resulting from support from this training grant. The assessment considered papers published between September 2010 and July 2022. We also quantified the number of publications and new research grants written and awarded to senior researchers and trainees involved in the EABRTM program, as well as the number of research administrators and trainees who received complementary training in biostatistics.

Improvements in *institutional capacity* were assessed as the number of postgraduate training programs designed or supported (master’s and PhD programs), the number of students who obtained a postgraduate diploma or degree, and the number of workshops, courses, and seminars delivered to trainees and the broader academic community. Research administration capacity development and *north-south* and *south-south collaboration* were evaluated according to the number of joint research and training projects written and implemented, and by the number of joint manuscripts published and abstracts presented in international meetings. Primary outcomes were also evaluated by using descriptive statistics.

## Results

Concerning postgraduate programs designed and supported, at UniLúrio, three master’s programs designed during the MEPI period received support for their implementation, MTMGH, MHPE, and MNFS. Between 2015 and 2018, 202 students entered these master’s programs. Of these, 152 (75.2%) obtained a postgraduate diploma on the basis of their first year of academic studies. A total of 89 (44.0%) completed the master’s degree [3]. Subsequently, an additional 24 students obtained postgraduate diplomas and 18 obtained master’s degrees from 2019 to 2021 (MHPE (9) and MNFS (9)). Ultimately 176 (87.1%) and 107 (53.0%) students obtained postgraduate diplomas and master’s degrees, respectively.

At UEM, the EABRTM program provided support for the design and initiation of the master’s program in Biosciences with three tracks: Parasitology, Microbiology and Neurosciences. Twenty-three students have been selected and enrolled in this program, with two of them supported by a scholarship through this grant. As for the other master’s programs, the available slots have first prioritized researchers and faculty members (11) from the institutions involved in this consortium [3].

The DPBPH was initiated in 2017 at the Faculty of Medicine, in 2018 at the Faculty of Sciences and in 2019 at the Faculty of Veterinary Science and was supported by this program through a number of above-mentioned short courses and seminars provided to students, mentors and the overall academic community (See Table 1). As of January 2022, a total of 31 students have been enrolled in this doctoral program, with 16 of them supported by other NIH training grants (the EABTM (D43 TW010568) (8); PALOP D43 TW009675-06 (4) and NIH PRISM D43TW009745-08 (4)). The remaining doctoral students are supported by other national and international funders.

**Table 1:** Short courses and number of participants developed and implemented by the EABRTM program.


COURSES	2016		2017		2018		2019		2020		TOTAL 2016–2020	
					
	NC	TP	NC	TP	NC	TP	NC	TP	NC	TP	NC	TP

Scientific manuscript writing	2	43	4	73	6	105	14	78	3	37	29	336

Quantitative research methods	1	17	3	63	4	94	1	10	1	15	10	199

Qualitative research methods	2	38	2	19	3	32	3	41	3	37	13	167

Good clinical pratices	–	–	2	30	5	105	1	25	–	–	8	160

Grant writing and management	1	20	3	40	4	60	–	–	–	–	8	120

Laboratory biosafety	–	–	2	20	2	20	4	25	–	–	8	65

Laboratory skills training	4	4	2	20	2	10	1	10	2	20	11	64

Bioethics and human subjects	2	137	2	50	2	125	3	75	3	75	12	462

RCR	2	15	2	22	2	50	3	60	3	75	12	222

Other courses*	2	117	1	25	2	125	2	50	1	30	8	347

Total	**16**	**391**	**23**	**362**	**32**	**726**	**32**	**374**	**16**	**289**	**119**	**2,142**


NC: Number of Courses; TP: Total participants. * These include the following: “Protocols and Ethics in Research” and “How to prepare scientific communications for conferences”.

With regard to the design, development and implementation of postgraduate short courses, during the study period, we organized 119 courses and seminars that benefitted 2,142 participants from over 14 institutions located in seven of Mozambique’s 11 provinces (Table 1). In addition, seven trainees were sponsored for short courses conducted outside Mozambique (Portugal, South Africa, Spain and the USA). These courses included the following fields: Research in Health Sciences; Educational Innovation; Rotavirus; Quantitative and Qualitative Research Methods; Molecular Biology; Biostatistics and Phylogenetic Analysis. All trainees also enrolled in NIH Human Subjects and RCR courses. A highly effective mentorship program was consolidated at UEM composed of senior faculty (8), three of whom trained in 2016 at the Glocal workshop in South Africa [23], as well as junior mentors (17).

The program exceeded its goal of 18 trainees (training of biomedical research leaders) and instead supported 39 trainees (17 junior mentors and 22 postgraduate students) at the master and PhD level. Thirteen of 22 trainees completed a master’s degree, as follows: UEM master’s in Public Health (n = 3), Masters in Biotechnology (1), UniLúrio MTMGH (5), and MHPE (4). One UniLúrio faculty member completed a PhD in Education Sciences at the Catholic University of Mozambique. Two trainees dropped-out and six are still in the program. Of those completing master’s degrees, four are now pursuing doctoral studies at UEM and one is doing so abroad. As of July, 2022, 35 manuscripts were published in peer-reviewed international journals, including articles from Mozambican trainees (16, 46%) and from senior faculty as a result of networking with international partners (19, 54%). Articles published in a national scientific journal (1) or by an open access publisher (1) and one book from the AFREhealth Governing Committee were not included in this tabulation.

**Figure 1:**
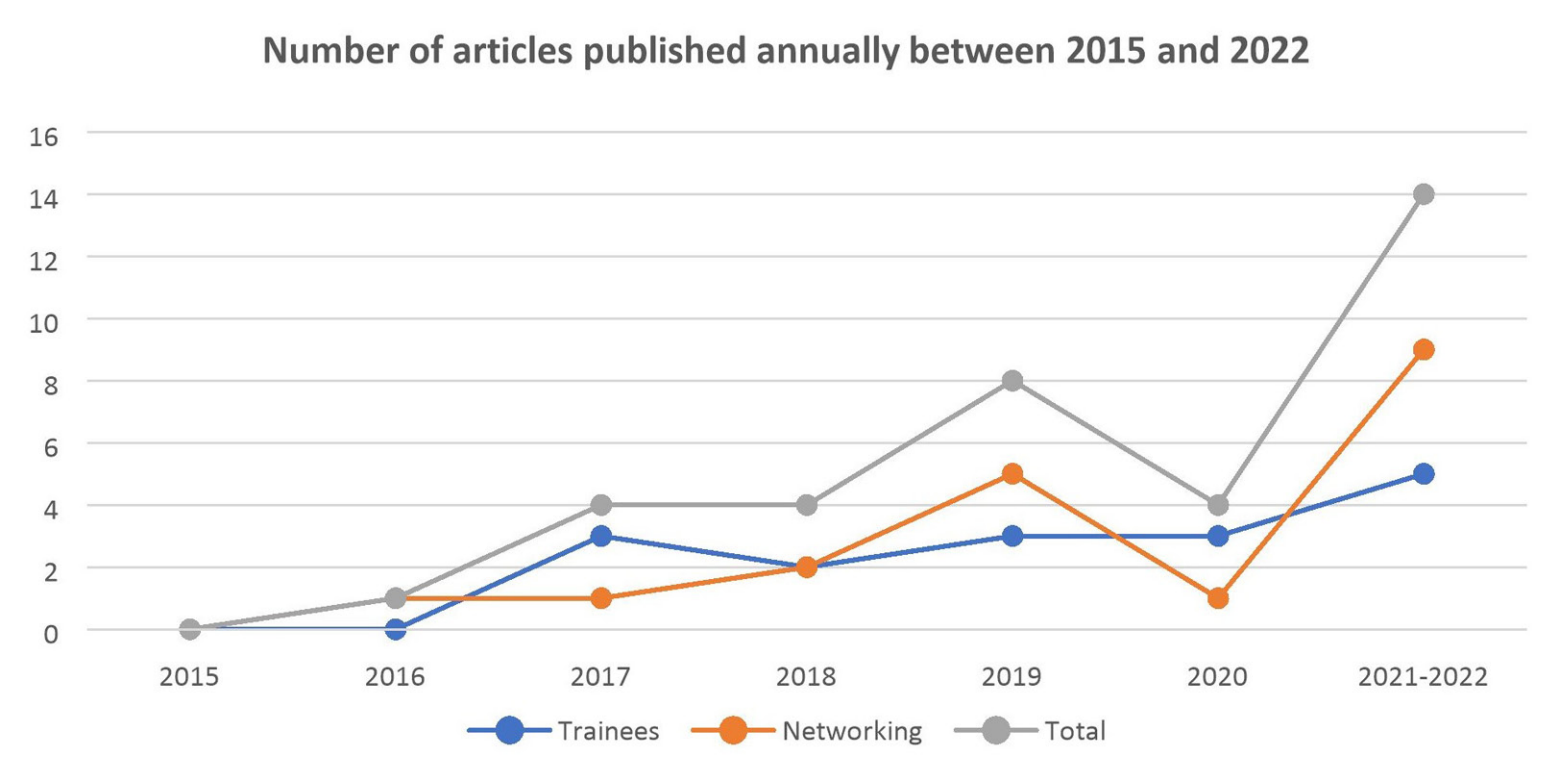
Number of articles published between 2015 and 2022.

Figure 1 illustrates the peer-reviewed publications by year derived from the project from 2015–2022.

Seventy-seven percent (27/35) of publications were first-authored by Mozambicans (Table 2 and Supplementary file 1). Last authors were predominantly foreigners (76%, n = 26/34) with Africans comprising 44% (15 out of 34). Foreign authors (172) were from across all continents, mostly from other countries from Africa (65%, n = 111), America (23%, n = 40), Europe (9%, n = 16) and Asia (3%, n = 5).

**Table 2:** Summary of characteristics, countries and institutions involved in the peer-reviewed scientific publications funded by EABRTM.


		ARTICLES FROM TRAINEES N(%)	ARTICLES FROM NETWORKING N(%)	TOTAL

**Number of authors**		55*	190*	237*

**Number of institutions involved**	Mozambican	9*	9*	12*

	Foreign	13*	81*	91*

**Number of countries**		9*	24*	28*

Mozambican first publication (PubMed)**		9/39 (23%)	0/36 (0%)	9/39 (23%)

**Mozambican first author**		16/16 (100%)	11/19 (58%)	27/35 (77%)

**Mozambican second author**		12/15 (80%)	6/17 (35%)	18/32 (56%)

**Mozambican third author**		9/13 (69%)	5/16 (31%)	14/29 (48%)

**Mozambican last author**		7/16 (44%)	1/18 (6%)	8/34 (24%)


* The percentage was not calculated because the numerator is the same as the denominator in these cases. ** By 39 trainees. *Note*: Most numbers do not result from the sum because there were common names in those groups.

Mozambican trainees and senior faculty actively participated in international scientific conferences and network meetings with presentation of 23 abstracts in total (See Supplementary file 2).

Concerning development of research administrative infrastructure, the EABRTM program trained 13 junior research administrators from MIHER (5), UEM (4), UniLúrio (2) and the INS (2) in research administration. Furthermore, five senior and junior MIHER research administrators participated in a total of 15 international courses and conferences organized by the International Society of Research Administrators (Supplementary file 2).

From 2015 until June 2021, MIHER supported the joint writing and submission of 72 proposals by senior faculty members and trainees. Of these, 59 (82%) were approved for funding by international agencies. These agencies were from the US (73%, n = 43), Europe (24%, n = 14) and Oceania (3%, n = 2) totaling $16,363,656.42, including the training grant discussed here ($3,205,000). Thirteen of our grant applications were either not awarded or are still awaiting a funding decision.

**Figure 2:**
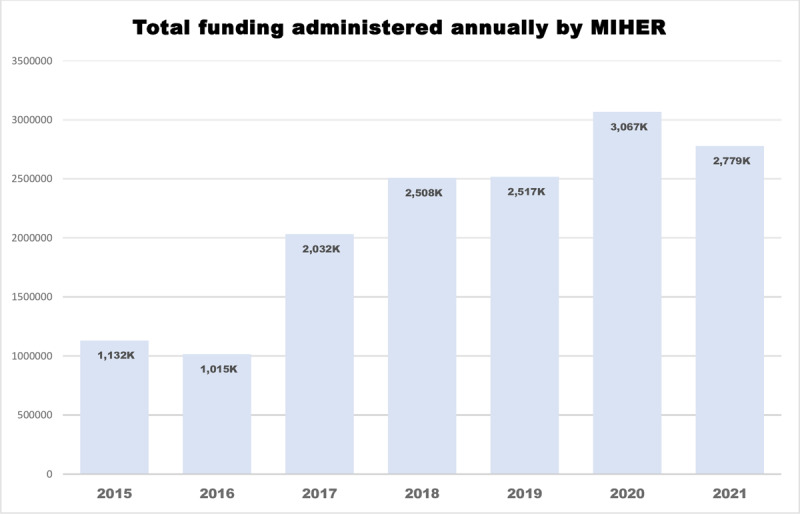
Total funding administered per year by MIHER.

Figure 2 shows the evolution of funding during the study period. The Fogarty International Center – NIH is the major contributor (28 awards, 47.6%) ($13,176,735.00), followed by the German Federal Ministry of Education and Research (BMBF) (3 awards, 5.1%) ($1,803,141.09) (Supplementary file 3). MIHER has secured 25 research awards, totaling an amount of $7,319,366.11 (25 projects), for the next years (until 2025) to support further research capacity building and the training of health professionals.

Information technology enhancements enabled file sharing amongst grant management officials within MIHER and partnering institutions, and launched the Asana application. This grant management software package allows MIHER grant management staff to manage projects, assign work to teammates, specify deadlines, and communicate about tasks directly in one tool. Additionally, as part of institutional networking, one of our senior researchers is part of the Training Advisory Committee for a research grant from the University of Nigeria.

A Biostatistics Unit led by two mid-career UEM faculty members with support from four trainees was established to provide support in basic data analysis using qualitative and quantitative methods.

**Figure 3:**
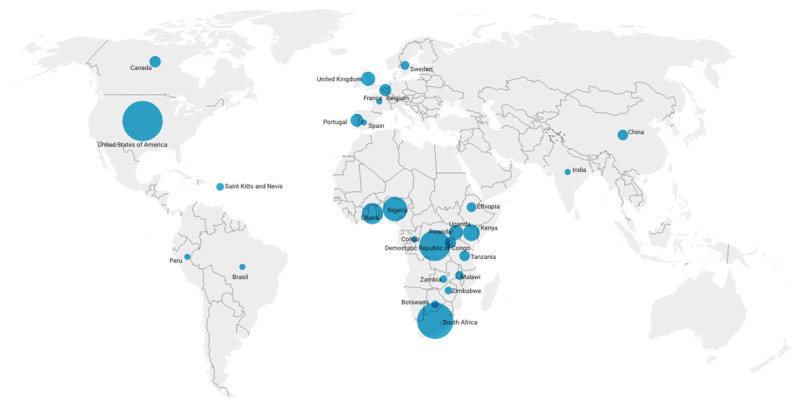
Countries involved in the north-south and south-south collaboration joint research and publications within the EABRTM. *Note*: Larger circles represent countries where there was a greater proportion of authorship mentions. The choropleth map was created in the following website: https://www.datawrapper.de.

North-south and south-south collaboration activities developed in this EABRTM program involved participation of 103 institutions from 28 countries. The majority of institutions involved in this research capacity building initiative were from Africa (62, 60%) followed by the American continent (27, 26%) mostly represented by USA (22, 82%) (Figure 3). Additional collaborations with institutions in Europe, Asia and Australia were also established (Supplementary file 1).

## Discussion

This manuscript summarizes activities and discusses the achievements and legacy resulting from the five-year funding period of the EABRTM program to train a new generation of Mozambican leaders in biomedical and behavioral research, develop and strengthen a research infrastructure, and foster north-south and south-south collaboration initiated during MEPI [12]. The EABRTM program exceeded targeted goals for the number of trainees by more than 100%. The depreciation of the Mozambican currency over the years, increased the exchange rate and amplified the impact of grant funds awarded in this currency.

The EABRTM program positioned Mozambican scientists as first authors in 77% of published manuscripts, reversing prior experiences where most publications from Mozambique were first-authored by researchers from high income countries [2,12,14,15,20]. In our previous study, we found that while a total of 202 manuscripts were published by 200 faculty members from the UEM Faculty of Medicine between 2001 and 2010, of those, Mozambican authors were the first authors in only 58 (29%) papers [12]. In addition, Mozambicans or Africans from other countries served as last authors of 44% (115/34) of EABRTM manuscripts. On the other hand, the record also reflects the chronic scarcity of national/African mentors [12,15]. The experiences and scientific knowledge generated at various scientific meetings also contributed to the design of policies and guidelines, helped to prioritize resource allocation, and attracted more investments to the health sector, thus contributing to the sustainable development of the country towards achievement of equity in global health, as widely documented in the literature [3,12,15,21,24,25,26,27,28,29,30,31,32,33,34,35]. Because peer-reviewed journal publication is a prolonged process, especially for researchers with less-developed writing skills [24], the number of publications listed does not fully reflect the impact of the present Fogarty training program, as some program participants are still pursuing studies, conducting research and writing manuscripts.

Contrary to traditional models of mentorship, and taking into account the scarcity of experienced role models, through the diverse courses, workshops and seminars we were able to create an environment in which trainees themselves could provide reciprocal mentorship [3,36,37]. This strengthened their own mentorship skills and laid foundations for successful mentorship: reciprocity, respectful relationships with clear expectations, interpersonal connections, and learner-focused goals [38]. This contributed to inter- and trans-disciplinary research and capacity building. Unlike in most traditional research capacity building initiatives, participants could choose research topics according to both their predilections and their mentors’ interest. By doing so, this fostered even greater enthusiasm towards their research [3,15,20].

The EABRTM program had additional indirect outcomes as some trainees’ research work involved the recruitment of patients in the hospitals, which in turn required the involvement of additional research team members, such as doctors, nurses and psychologists. Thus, new potential scientists were exposed to, initiated in, and motivated towards scientific research activities.

This Fogarty training grant fostered north-south and south-south collaboration among MEPI schools and their US counterparts through the AFREhealth forum and MEPI D43 networking meetings, CUGH conferences, and the European and Developing Countries Clinical Trials Partnership (EDCTP) forum meetings and allowed joint research and publications on the most pressing health issues related to the African continent, and Mozambique in particular [26,21,27,28,29,30,31,32,34,35].

Retention of trainees was excellent. All trainees, including mid-career and senior researchers involved in this research capacity building program, have remained at their home institutions, and are now supporting more junior colleagues seeking to initiate investigative careers. This can, in part, be attributed to scholarship support provided to trainees and monetary incentives given to mentors to buy-out their time for research. Otherwise, they would have been unable to withdraw from other activities required to support basic income needs. Indeed, most brain drain in Mozambique is internal, with individuals moving from the public sector to NGOs. Other factors contributing to retention were the improvement of working conditions and the contributions to career development offered by the program [11,12]. It is well-documented that faculty satisfaction and motivation are key factors to avoid brain drain. Between 65 and 70% of African scientists leave their countries of birth to pursue education and employment, mostly in high-income countries, in order to advance their careers and increase income [12,15,25,39].

The administrative research infrastructure initiated by MEPI through MIHER has been consolidated [12], as demonstrated by the growing number of grant proposals that were successfully written and submitted (72) or awarded and implemented (59), compared with what was achieved previously and during the MEPI period. Compared to 2010–2014, MIHER increased its capacity from 22 projects, totaling $12,251,780, written and awarded to 59 projects, totaling $16,363,656.42, for 2015–2020, despite a reduction in 2021 due to the prioritization of international agencies on COVID-related activities. The increased research administration capacity is contributing to the sustainability of MIHER and the long-term collaborations initiated during MEPI [2]. Multiple Mozambican research administrators are now able to deal with the administrative requirements for NIH and other US federal funds as well as funds from other international agencies. By building this infrastructure, we have addressed a very important constraint faced not only within Mozambique, but more broadly by the African research community [15]. While we have been exposed to several research capacity building initiatives in Mozambique with support from various donors and funding agencies, to the best of our knowledge, the MEPI and related NIH initiatives (including this one) are the first that simultaneously addressed support in specific scientific areas as well as in training of research administration, including the creation of the research support center, MIHER.

Biomedical courses in Mozambique and sub-Saharan Africa, more broadly, offer biostatistics as an elementary discipline, and fail to provide students with comprehensive knowledge in research study design and statistical analyses. Few universities in Africa provide advanced training in statistics and even fewer are linked to programs that emphasize biomedical research applications [40,41,42,43], further limiting the ability of junior researchers to properly design and perform adequate data analyses in biomedical research. Sub-Saharan African countries contribute only 2% of the world’s published research, despite the fact that they bear 25% of the world’s disease burden; rejection of submitted papers is largely based on poor study design and statistical methods [15,43]. The few existing biostatisticians in Mozambique are absorbed by the private sector or NGOs where they receive better compensation than in the public sector. By training our biomedical researchers in biostatistics and creating a biostatistics unit we are ensuring that they will help other researchers in the analysis of complex data and be retained in our academic institutions as they develop their own specific areas in biomedical research.

Taken together, our Mozambican-led program supports direct engagement with major funding agencies, and shifts from traditional models of north-south collaboration to one led by Africans who are better positioned to identify the most compelling local research needs. This approach creates a greater sense of ownership and leadership and thus supports future sustainability [3,12,15,25]. Similar outcomes were achieved by other research capacity building initiatives such as Heredity and Health in Africa (H3Africa), the Wellcome Trust, the National Academy of Sciences, the Better Health Information for Better Health Policy (INDEPTH) network, the Consortium for Health Systems Policy Analysis in Africa (CHEPSAA), and the partnership between Makerere University and Karolinska Institutet, amongst others [3,15].

Despite our progress and achievements, we are still challenged by a shortage of skilled mentors, as the few existing ones are overwhelmed. However, our mentorship experience in using students to mentor their counterparts (peer mentoring) has proven to be effective, similar to initiatives in other countries sharing the same human resource limitations [3,36,37]. Limited fluency in English has traditionally challenged Mozambican scientists preparing manuscripts and applying for research grants. This limitation was partially overcome by joint manuscript and grant writing with our English-speaking partners, by encouraging our trainees to take English language courses and by intensive interactions in joint research efforts, as well short courses focused on English scientific writing terminology [3,13].

More recently, the unexpected and unprecedented coronavirus 2019 (COVID-19) pandemic had a negative impact on our productivity secondary to lockdowns and several other imposed restrictions that affected or slowed down our progress. The situation significantly affected the importation of supplies and reagents for the work of our trainees and prevented travel to and from US to participate in various training courses and laboratory work. Physical distancing imposed for mitigation of the spread of SARS-CoV-2 also affected recruitment of patients for studies. Although distance learning and zoom meetings kept activities from grinding to a halt, they are not effective substitutes for in person activities. Quarantines, isolations and psychological consequences faced by our trainees as they were infected or had to deal with infected or ill family members were also highly disruptive. The initiation of the master’s program in Biosciences was delayed, but with increasing use of remote communication technologies we ultimately were able to initiate the program despite being in the midst of a third wave of the COVID-19 pandemic. Indeed, in order to address the unexpected pandemic, many senior African academicians and scholars were forced to shift their routine research focus to COVID-related topics.

Experiences gained from this program extend well beyond biomedical research to other areas of scholarship in sub-Saharan countries and to the world in general [28,29,30,32,44]. We have learned that such investments should be continued in the future so that trainees and midcareer faculty members can develop their careers and can mentor the next generations of researchers. This momentum we developed under EABRTM will be sustained by funded research grants enabled by the training and research infrastructure developed with EARBTM support.

## Conclusion

Although Mozambique has been exposed to many capacity-building collaborations that have taken different approaches, the EABRTM program was conducted in a systematic way with focus on country priorities, as well as the empowerment of local scientists and institutions, and has made a considerable contribution to north-south and south-south collaboration. We have trained a new generation of biomedical researchers equipped with key didactic and research skills that ultimately will improve the working environment, increase career satisfaction, and build institutional capacity with retention of those trained within public sector universities and institutions. Sustainability of our strategy has been demonstrated by the number of proposals written and awarded and by the fact that it is embedded in multiple critical interconnected programs and resources. Collaborations established between our trainees and faculty members with counterparts at UCSD and African peer institutions will enable EARBTM’s reach to extend well beyond the funding period. The programs described here fostered learning, collaboration and resource-sharing among national and international institutions, and contributed to an increase in scientific output as reflected by publications and grants awarded.

## Additional Files

The additional files for this article can be found as follows:

10.5334/aogh.3684.s1 Supplementary File 1. Articles published through the EABRTM and statistics regarding the authorship, institutions and countries.

10.5334/aogh.3684.s2 Supplementary File 2. Description of the participation of trainees, mentors and research administrators in international scientific meetings and courses.

10.5334/aogh.3684.s3 Supplementary File 3. Funding agencies of the 59 grants administered by MIHER from 2015 to 2021.
