# Supplementary material for: Fostering Sustainable Biomedical Research Training in Mozambique: A Spin-Off of the Medical Education Partnership Initiative
Source: Ann Glob Health. 2022 Aug 2;88(1):65. doi: 10.5334/aogh.3684 (PMC9354555; doi:10.5334/aogh.3684)
Supplement: Supplementary File 1. — Articles published through the EABRTM and statistics regarding the authorship, institutions and countries. [file agh-88-1-3684-s1.pdf]

Supplemental file 1: Articles published through the EABRTM and statistics regarding the authorship, institutions and countries.

A. Articles from trainees

1. Cerveja, B. Z., Tucuzo, R. M., Madureira, A. C., Nhacupe, N., Langa, I. A., Buene, T., Banze, L., Funzamo, C., & Noormahomed, E. V. (2017). Prevalence of Intestinal Parasites Among HIV Infected and HIV Uninfected Patients Treated at the 1° De Maio Health Centre in Maputo, Mozambique. EC microbiology, 9(6), 231–240.
2. Matsimbe, A. M., Magaia, V., Sanches, G. S., Neves, L., Noormahomed, E., Antunes, S., & Domingos, A. (2017). Molecular detection of pathogens in ticks infesting cattle in Nampula province, Mozambique. Experimental & applied acarology, 73(1), 91–102. https://doi.org/10.1007/s10493-017-0155-5.
3. Chabite, I. T., Lei, Z., Ningning, Y., Qiang, F., & Haiye, Y. (2017). Mode of Managing Nutrient Solution Based on N Use Efficiency for Lettuce (*Lactuca sativa* L.). Journal of food science and engineering, 7, 29–37. https://doi.org/10.17265/2159-5828/2017.01.003.
4. Comia, I., Madureira, A. C., Schooley, R. T., Vieira, M. L., & Noormahomed, E. V. (2018). Molecular Detection of *Leptospira* spp. in Rodents Trapped in the Mozambique Island City, Nampula Province, Mozambique. EC microbiology, 14(12), 813–821.
5. Saldanha, V., Saldanha, G., Reys, R. P., Benson, C. A., & Noormahomed, E. V. (2018). Neurocysticercosis in Child Bearing Women: An Overlooked Condition in Mozambique and a Potentially Missed Diagnosis in Women Presenting with Eclampsia. EC microbiology, 14(11), 736–740.
6. Cossa, M., & Robinson, T. D. (2019). Intrapericardial diaphragmatic hernia in a 6-month-old girl: A case report and review of the literature. International journal of surgery case reports, 60, 49–52. https://doi.org/10.1016/j.ijscr.2019.05.049.
7. Tchamo, C. C., De Rugeriis, M., & Noormahomed, E. V. (2019). Occurrence of feline immunodeficiency virus and feline leukaemia virus in Maputo city and province, Mozambique: a pilot study. JFMS open reports, 5(2), 2055116919870877. https://doi.org/10.1177/2055116919870877.
8. Nhancupe, N., Noormahomed, E. V., Afonso, S., Svard, S., & Lindh, J. (2019). Further evaluation of recombinant Tsol-p27 by enzyme-linked immunoelectrotransfer blot for the serodiagnosis of cysticercosis in pigs from Mozambique. Parasites & vectors, 12, 564. https://doi.org/10.1186/s13071-019-3816-x.
9. Miambo, R. D., Afonso, S.M.S., Noormahomed, E. V., Pondja, A., & Mukaratirwa, S. (2020). Echinococcosis in humans and animals in Southern Africa Development Community countries: A systematic review. Food and waterborne parasitology, 20, e00087. https://doi.org/10.1016/j.fawpar.2020.e00087
10. Hlashwayo, D. F., Sigaúque, B., & Bila, C. G. (2020). Epidemiology and antimicrobial resistance of *Campylobacter* spp. in animals in Sub-Saharan Africa: A systematic review. Heliyon, 6(3), e03537. https://doi.org/10.1016/j.heliyon.2020.e03537.
11. Manuel, L., Bechel, A., Noormahomed, E. V., Hlashwayo, D. F., & Madureira, M. (2020). Ethnobotanical study of plants used by the traditional healers to treat malaria in Mogovolas district, northern Mozambique. Heliyon, 6(12), e05746. https://doi.org/10.1016/j.heliyon.2020.e05746.
12. Banze, L., Madureira, A.C., Zacarias, B.C., Nhacupe, N., Mascaro-Lazcano, C., Benson, C.A., Schooley, R.T. & Noormahomed, E. (2021). Coinfection of HIV-1 with *Schistosoma* spp. and with Intestinal Parasites in Patients Attending Boane Health Center, Maputo Province, Mozambique. EC Microbiology, 17.5: 3-16.
13. Cossa, M., Rose, J., Berndtson, A. E., Noormahomed, E., & Bickler, S. W. (2021). Assessment of Surgical Care Provided in National Health Services Hospitals in Mozambique: The Importance of Subnational Metrics in Global Surgery. World journal of surgery, 45(5), 1306–1315. https://doi.org/10.1007/s00268-020-05925-0.
14. Sousa, I.M, Zucula, L., Nhancupe, N., Banze, L., Zacarias, B. & Noormahomed, E.V. (2021). Assessment of Parasitic Contamination of Lettuce and Cabbages Sold in Selected Markets in Maputo City, Mozambique. EC microbiology, 17.6 (2021): 27-37.
15. Miambo, R.D., Afonso, S., Noormahomed, E. V., Malatji, M. P. & Mukaratirwa, S. (2022). Prevalence and molecular characterization of cystic hydatidosis in livestock slaughtered in southern Mozambique. Journal of Parasitic Diseases, 46, 186–195. https://doi.org/10.1007/s12639-021-01434-6.
16. Jethá, E., Keygnaert, I., Martins, E., Sidat, M., & Roelens, K. (2021). Domestic Violence In Mozambique: From policy to practice. BMC Public Health, 21, 772. https://doi.org/10.1186/s12889-021-10820-x.
17. Zavale, B.L., Spencer, E.A., Guilundo, C. Schooley, R., Patel, S., Noormahomed, E.V. & Mocumbi, A.O. (2019). Closing the Gaps on Medical Education in Low-Income Countries Through Information &

Communication Technologies: The Mozambique Experience. Biomed J Sci & Tech Res, 16(4)-2019. BJSTR. MS.ID.002875.

18. Botas, L., Amade, N., Augusto, Â., Mabunda, N. & de Deus, N. (2016). [Clinical and Laboratory Profile of Patients Referred for the Screening of Human Lymphotropic Virus Type 1 and 2 T cells] *Perfil Clínico-Laboratorial dos Pacientes Referenciados para o Rastreio do Vírus Linfotrópicos Humanos de células T Tipo 1 e 2*. Revista Moçambicana de Ciências de Saúde (3)1: 14-21.

Note: Zavale et al. (2019) and Botas et al. (2016) are not included in the peer-reviewed manuscripts statistics.

B. Articles from the networking *(north-south and south-south collaboration)*

1. Noormahomed, E. V., Akrami, K., & Mascaró-Lazcano, C. (2016). Onchocerciasis, an undiagnosed disease in Mozambique: identifying research opportunities. Parasites & vectors, 9, 180. https://doi.org/10.1186/s13071-016-1468-7.
2. Omaswa, F., Kiguli-Malwadde, E., Donkor, P., Hakim, J., Derbew, M., Baird, S., Frehywot, S., Gachuno, O. W., Kamiza, S., Kibwage, I. O., Mteta, K. A., Mulla, Y., Mullan, F., Nachega, J. B., Nkomazana, O., Noormohamed, E., Ojoome, V., Olalaye, D., Pillay, S., Sewankambo, N. K., de Villiers, M. (2017). Medical Education Partnership Initiative gives birth to AFREhealth. The Lancet. Global health, 5(10), e965–e966. https://doi.org/10.1016/S2214-109X(17)30329-7.
3. Noormahomed, E. V., Mocumbi, A. O., Ismail, M., Carrilho, C., Patel, S., Nguenha, A., Badaro, R., Bickler, S., Benson, C. A., & Schooley, R. T. (2018). The Medical Education Partnership Initiative Effect on Increasing Health Professions Education and Research Capacity in Mozambique. Annals of global health, 84(1), 47–57. https://doi.org/10.29024/aogh.14.
4. Omaswa, F., Kiguli-Malwadde, E., Donkor, P., Hakim, J., Derbew, M., Baird, S., Frehywot, S., Gachuno, O. W., Kamiza, S., Kibwage, I. O., Mteta Kien, A., Mulla, Y., Mullan, F., Nachega, J. B., Nkomazana, O., Noormohamed, E., Ojoome, V., Olalaye, D., Pillay, S., Sewankambo, N. K. & De Villiers, M. (2018). The Medical Education Partnership Initiative (MEPI): Innovations and Lessons for Health Professions Training and Research in Africa. Annals of global health, 84(1), 160–169. https://doi.org/10.29024/aogh.8.
5. Noormahomed, E., Williams, P., Lescano, A. G., Raj, T., Bukusi, E. A., Schooley, R. T., & Cohen, C. R. (2019). The Evolution of Mentorship Capacity Development in Low- and Middle-Income Countries: Case Studies from Peru, Kenya, India, and Mozambique. The American journal of tropical medicine and hygiene, 100(1_Suppl), 29–35. https://doi.org/10.4269/ajtmh.18-0560.
6. Carrilho, C., Ismail, M., Lorenzoni, C., Fernandes, F., Alberto, M., Akrami, K., Funzamo, C., Lunet, N., & Schmitt, F. (2019). Fine needle aspiration cytology in Mozambique: Report of a 15-year experience. Diagnostic cytopathology, 47(3), 166–171. https://doi.org/10.1002/dc.24062.
7. Cunha, L., Carrilho, C., Bhatt, N., Loforte, M., Maueia, C., Fernandes, F., Guisseve, A., Mbofana, F., Maibaze, F., Mondlane, L., Ismail, M., Dimande, L., Machatine, S., Lunet, N., Liu, Y. T., Gudo, E. S., & Pineau, P. (2019). Hepatocellular carcinoma: Clinical-pathological features and HIV infection in Mozambican patients. Cancer treatment and research communications, 19, 100129. https://doi.org/10.1016/j.ctarc.2019.100129.
8. Noormahomed, E. V., & Mascaró-Lazcano, C. (2019). Onchocerciasis in Mozambique: An Unknown Condition for Health Professionals. EC microbiology, 15(3), 160–167.
9. Noormahomed, E. V. (2019). Opportunities and Challenges for Strengthening Biomedical Research in Sub Saharan Countries: The Mozambique Experience. EC Microbiology,15(9): 1049-1051.
10. Carrilho, C., Miu, C., Kim, Y., Karki, S., Balmaceda, A., Challa, B., Diamond, S., Monteiro, E., Marole, E., Lorenzoni, C., Zambujo, Y., Liu, Y. T., Schooley, R. T., & Lin, J. H. (2020). p16 Expression Correlates with Invasive Ocular Surface Squamous Neoplasms in HIV-Infected Mozambicans. Ocular oncology and pathology, 6(2), 123–128. https://doi.org/10.1159/000502096.
11. Nachega, J. B., Grimwood, A., Mahomed, H., Fatti, G., Preiser, W., Kallay, O., Mbala, P. K., Muyembe, J. T., Rwagasore, E., Nsanzimana, S., Ngamije, D., Condo, J., Sidat, M., Noormahomed, E. V., Reid, M., Lukeni, B., Suleman, F., Mteta, A., & Zumla, A. (2021). From Easing Lockdowns to Scaling Up Community-based Coronavirus Disease 2019 Screening, Testing, and Contact Tracing in Africa-Shared Approaches, Innovations, and Challenges to Minimize Morbidity and Mortality. Clinical infectious diseases : an official publication of the Infectious Diseases Society of America, 72(2), 327–331. https://doi.org/10.1093/cid/ciaa695.
12. Ismail, M. R., Noormahomed, E. V., Lawicki, S., & Eichbaum, Q. (2021). Survey of Clinical and Anatomic Pathology Laboratory Infrastructure in Mozambique. American journal of clinical pathology, 156(5), 810–817. https://doi.org/10.1093/ajcp/aqab026.
13. Noormahomed, E.V., Nhancupe, N., Mufume, J., Schooley, R.T., Foyaca-Sibat, H., Benson, C.A (2021). Neurocysticercosis in Epileptic Children: an Overlooked Condition in Mozambique, Challenges in Diagnosis, Management and Research Priorities. EC Microbiology. 17.6 (2021): 49-56.
14. Noormahomed, E. V., Mandane, A., Cuambe, A., Rodrigues, M. A., Noormahomed, S., Carrilho, C., Mocumbi, A. O., Ali, M., Vintuar, P., Ismail, M., Guilundo, C., Bickler, S., Benson, C. A., Ferrão, J. L., & Schooley, R. T. (2021). Design and Implementation of Postgraduate Programs in Health in a Resource-

Limited Setting in Mozambique (The Lúrio University). Advances in medical education and practice, 12, 399–412. https://doi.org/10.2147/AMEP.S291364.

15. Nachega, J. B., Sam-Agudu, N. A., Masekela, R., van der Zalm, M. M., Nsanzimana, S., Condo, J., Ntoumi, F., Rabie, H., Kruger, M., Wiysonge, C. S., Ditekemena, J. D., Chirimwami, R. B., Ntakwinja, M., Mukwege, D. M., Noormahomed, E., Paleker, M., Mahomed, H., Tamfum, J. M., Zumla, A., & Suleman, F. (2021). Addressing challenges to rolling out COVID-19 vaccines in African countries. The Lancet. Global health, 9(6):e746-e748. https://doi.org/10.1016/S2214-109X(21)00097-8.

16. Sam-Agudu, N. A., Rabie, H., Pipo, M. T., Byamungu, L. N., Masekela, R., van der Zalm, M. M., Redfern, A., Dramowski, A., Mukalay, A., Gachuno, O. W., Mongweli, N., Kinuthia, J., Ishoso, D. K., Amoako, E., Agyare, E., Agbeno, E. K., Jibril, A. M., Abdullahi, A. M., Amadi, O., Umar, U. M., Ayele, B. T., Machekano, R. N., Nyasulu, P. S., Hermans, M. P., Otshudiema, J.O., Bongo-Pasi Nswe, C., Kayembe, J. N., Mbala-Kingebeni, P., Muyembe-Tamfum, J. J., Aanyu, H.T., Musoke, P., Fowler, M.G., Sewankambo, N., Suleman, F., Adejumo, P., Tsegaye, A., Mteta, A., Noormahomed, E. V., Deckelbaum, R. J., Zumla, A., Mavungu Landu, D. J., Tshilolo, L., Zigabe, S., Goga, A., Mills, E. J., Umar, L. W., Kruger, M., Mofenson, L. M., Nachega, J. B.; for investigators in the AFREhealth COVID-19 Research Collaboration on Children and Adolescents (2021). The Critical Need for Pooled Data on Coronavirus Disease 2019 in African Children: An AFREhealth Call for Action through Multicountry Research Collaboration. Clinical infectious diseases : an official publication of the Infectious Diseases Society of America, 73(10), 1913–1919. https://doi.org/10.1093/cid/ciab142.

17. Nachega, J. B., Sam-Agudu, N. A., Machekano, R. N., Rosenthal, P. J., Schell, S., de Waard, L., Bekker, A., Gachuno, O.W., Kinuthia, J., Mwongeli, N., Budhram, S., Vannevel, V., Somapillay, P., Prozesky, H. W., Taljaard, J., Parker, A., Agyare, E., Opoku, A. B., Makarfi, A. U., Abdullahi, A. M., Adirieje, C., Ishoso, D. K., Pipo, M. T., Tshilanda, M. B., Nswe, C. B.-P., Ditekemena, J., Sigwadhi, L. N., Nyasulu, P. S. , Hermans, M. P., Sekikubo, M., Musoke, P., Nsereko, C., Agbeno, E. K., Yeboah, M. Y., Umar, L. W., Ntakwinja, M., Mukwege, D. M., Birindwa, E. K., Mushamuka, S. Z., Smith, E. R., Mills, E. J., Otshudiema, J. O., Mbala-Kingebeni, P., Tamfum, J-J. M., Zumla, A., Tsegaye, A., Mteta, A., Sewankambo, N. K., Suleman, F., Adejumo, P., Anderson, J. R., Noormahomed, E. V., Deckelbaum, R. J., Stringer, J. S. A., Mukalay, A., Taha, T. E., Fowler, M. G., Wasserheit, J. N., Masekela, R., Mellors, J. W., Siedner, M. J., Myer, L., Kengne, A-P., Yotebieng, M., Mofenson, L.M., Langenegger, E., for the AFREhealth Research Collaboration on COVID-19 and Pregnancy (2022). Severe Acute Respiratory Syndrome Coronavirus 2 (SARS-CoV-2) Infection and Pregnancy in Sub-Saharan Africa: A 6-Country Retrospective Cohort Analysis, Clinical Infectious Diseases, ciac294, https://doi.org/10.1093/cid/ciac294.

18. Adejumo, P. O., Nawagi, F., Kolawole, I.O., Ismail, M.R., Mukalay, A.W., Nabirye, R.C., Kazembe, A., Ojo, I.O., Adejumo, A.O., Nachenga, J.B., Suleman, F., Sewankambo, N.K., Okanlawon, F.A. & Noormahomed, E.V. (2021). Knowledge, preparedness, and attitude towards COVID-19 among health profession students in Sub-Saharan Africa: A cross-sectional survey. IJID Regions 1 (2021): 150-158. https://doi.org/10.1016/j.ijregi.2021.10.010.

19. Noormahomed, E. V. (2021). Chapter 6: Highlights of Past, Ongoing and Future Research Work: Perspective on TB, Malaria and NCDs. In P.O. Adejumo (Ed.). Transforming the Medical / Nursing Education Partnership Initiative into the African Forum for Research and Education in Health (pp. 67-77). Nigeria: Royalbird Books Ltd.

20. Nachega, J. B., Sam-Agudu, N. A., Machekano, R. N., Rabie, H., van der Zalm, M. M., Redfern, A., Dramowski, A., O'Connell, N., Pipo, M. T., Tshilanda, M. B., Byamungu, L. N., Masekela, R., Jeena, P. M., Pillay, A., Gachuno, O. W., Kinuthia, J., Ishoso, D. K., Amoako, E., Agyare, E., Agbeno, E. K., Martyn-Dickens, C., Sylverken J., Enimil, A., Jibril, A. M., Abdullahi, A. M., Amadi, O., Umar, U. M., Sigwadhi, L. N., Hermans, M. P., Otokoye, J. O., Mbala-Kingebeni, P., Muyembe-Tamfum, J. J., Zumla, A., Sewankambo, N. K., Aanyu, H. T., Musoke, P., Suleman, F., Adejumo, P., Noormahomed, E. V., Deckelbaum, R. J., Fowler, M. G., Tshilolo, L., Smith, G., Mills, E. J., Umar, L. W., Siedner, M. J., Kruger, M., Rosenthal, P. J., Mellors, J.W., Mofenson, L. M.; African Forum for Research and Education in Health (AFREhealth) COVID-19 Research Collaboration on Children and Adolescents (2022). Assessment of Clinical Outcomes Among Children and Adolescents Hospitalized With COVID-19 in 6 Sub-Saharan African Countries. JAMA pediatrics, 176(3), e216436. https://doi.org/10.1001/jamapediatrics.2021.6436.

D. Proportions and number of Mozambican and foreign authors in different positions of authorship in peer-reviewed publications

	Mozambican	Foreign
**Total authors***		
Total (Trainees and networking)	65/237 (27%)	172/237 (73%)
Trainees	33/55 (60%)	22/55 (40%)
Networking	36/190 (19%)	154/190 (81%)
**First authors**		
Total (Trainees and networking)	27/35 (77%)	8/35 (23%)
Trainees	16/16 (100%)	0/16 (0%)
Networking	11/19 (58%)	8/19 (42%)
**Second authors**		
Total (Trainees and networking)	18/32 (56%)	14/32 (44%)
Trainees	12/15 (80%)	3/15 (20%)
Networking	6/17 (35%)	11/17 (65%)
**Third authors**		
Total (Trainees and networking)	14/29 (48%)	15/29 (52%)
Trainees	9/13 (69%)	4/13 (31%)
Networking	5/16 (31%)	11/16 (69%)
**Last authors**		
Total (Trainees and networking)	8/34 (24%)	26/34 (76%)
Trainees	7/16 (44%)	9/16 (56%)
Networking	1/18 (6%)	17/18 (94%)

* The sum of the total authors does not result in the same number because there were common authors in these groups

E. Number of institutions and countries involved in the publications supported by the EABRTM.

E.1. Institutions

	Articles from trainees	Number of mentions	%
**1.**	UEM - Mozambique	44	34.6%
**2.**	MIHER - Mozambique	23	18.1%
**3.**	UCSD - USA	18	14.2%
**4.**	Jilin University - China	5	3.9%
**5.**	University of KwaZulu-Natal - South Africa	5	3.9%
**6.**	IHMT Universidade Nova de Lisboa - Portugal	4	3.1%
**7.**	UniLúrio - Mozambique	4	3.1%
**8.**	Ghent University - Belgium	3	2.4%
**9.**	Hospital Central de Quelimane - Mozambique	3	2.4%
**10.**	Uppsala University - Sweden	3	2.4%
**11.**	Centro de Biotecnologia - Mozambique	2	1.6%
**12.**	Hospital Central de Maputo - Mozambique	2	1.6%
**13.**	Ross University School of Veterinary Medicine - Saint Kitts and Nevis	2	1.6%
**14.**	Albany Medical Center - USA	1	0.8%
**15.**	CISM - Mozambique	1	0.8%
**16.**	INS - Mozambique	1	0.8%
**17.**	Memorial Sloan Kettering Cancer Center - USA	1	0.8%
**18.**	MISAU - Mozambique	1	0.8%
**19.**	South African National Biodiversity Institute – South Africa	1	0.8%
**20.**	Universidad de Granada - Spain	1	0.8%
**21.**	University of Coimbra - Portugal	1	0.8%
**22.**	University of Pretoria - South Africa	1	0.8%
	Total	***127***	***100.0%***
	**Articles from networking**	**Number of mentions**	**%**
**1.**	Stellenbosch University - South Africa	44	9.1%
**2.**	UEM FoM - Mozambique	36	7.5%
**3.**	UCSD - USA	32	6.6%
**4.**	Hospital Central de Maputo - Mozambique	24	5.0%
**5.**	University of KwaZulu Natal - South Africa	17	3.5%
**6.**	MIHER - Mozambique	16	3.3%
**7.**	University of Kinshasa - DR Congo	15	3.1%
**8.**	Johns Hopkins University - USA	11	2.3%
**9.**	Ahmadu Bello University - Nigeria	10	2.1%
**10.**	University of Cape Coast - Ghana	10	2.1%
**11.**	University of Ibadan - Nigeria	10	2.1%
**12.**	National Institute of Biomedical Research - DR Congo	9	1.9%
**13.**	University of Nairobi - Kenya	9	1.9%
**14.**	Cape Coast Teaching Hospital - Ghana	8	1.7%
**15.**	University of Pittsburgh - USA	8	1.7%
**16.**	Ahmadu Bello University Teaching Hospital - Nigeria	7	1.5%
**17.**	George Washington University - USA	7	1.5%
**18.**	African Centre for Global Health and Social Transformation - Uganda	6	1.2%
**19.**	Monkole Hospital Center - DR Congo	6	1.2%
**20.**	UniLúrio - Mozambique	6	1.2%
**21.**	INS - Mozambique	5	1.0%
**22.**	Institute of Human Virology - Nigeria	5	1.0%
**23.**	Kenyatta National Hospital - Kenya	5	1.0%
**24.**	Kilimanjaro Christian Medical University College - Tanzania	5	1.0%
**25.**	Université Notre-Dame du Kasayi - DR Congo	5	1.0%
**26.**	University College London - UK	5	1.0%
**27.**	University of California, San Francisco - USA	5	1.0%
**28.**	Addis Ababa University - Ethiopia	4	0.8%
**29.**	Asokoro District Hospital - Nigeria	4	0.8%
**30.**	Komfo Anokye Teaching Hospital - Ghana	4	0.8%
**31.**	Kwame Nkrumah University of Science and Technology - Ghana	4	0.8%
**32.**	Université Catholique de Bukavu - DR Congo	4	0.8%
**33.**	Université Evangelique en Afrique - DR Congo	4	0.8%
**34.**	University of Lubumbashi - DR Congo	4	0.8%
**35.**	University of Maryland - USA	4	0.8%
**36.**	Cliniques Universitaires St-Luc - Belgium	3	0.6%
**37.**	Cytel - Canada	3	0.6%
**38.**	Elizabeth Glaser Pediatric AIDS Foundation - USA	3	0.6%
**39.**	Hôpital Provincial Général de Référence de Bukavu - DR Congo	3	0.6%
**40.**	McMaster University - Canada	3	0.6%
**41.**	MISAU - Mozambique	3	0.6%
**42.**	Rwanda Biomedical Center - Rwanda	3	0.6%

**43.**	South African Medical Research Council - South Africa	3	0.6%
**44.**	Tygerberg Teaching Hospital - South Africa	3	0.6%
**45.**	Universidade do Porto - Portugal	3	0.6%
**46.**	Université Moderne de Kinkole - DR Congo	3	0.6%
**47.**	University College London Hospitals - UK	3	0.6%
**48.**	University of Malawi - Malawi	3	0.6%
**49.**	University of Pretoria - South Africa	3	0.6%
**50.**	World Health Organization Health Emergencies Program COVID-19 Response - DR Congo	3	0.6%
**51.**	Harvard Medical School - USA	2	0.4%
**52.**	Kheth'Impilo AIDS Free Living - South Africa	2	0.4%
**53.**	Mbarara University of Science and Technology - Uganda	2	0.4%
**54.**	NIHR Biomedical Research Centre - UK	2	0.4%
**55.**	Official University of Mbuji-Mayi - DR Congo	2	0.4%
**56.**	Tulane University - USA	2	0.4%
**57.**	University of Botswana - Botswana	2	0.4%
**58.**	University of Cape Town - South Africa	2	0.4%
**59.**	University of Rwanda - Rwanda	2	0.4%
**60.**	University of Washington - USA	2	0.4%
**61.**	University of Zambia - Zambia	2	0.4%
**62.**	University of Zimbabwe - Zimbabwe	2	0.4%
**63.**	Western Cape Department of Health - South Africa	2	0.4%
**64.**	Cliniques Universitaires de Lubumbashi - DR Congo	1	0.2%
**65.**	Entebbe Regional Reference Hospital - Uganda	1	0.2%
**66.**	Federal University of Bahia - Brazil	1	0.2%
**67.**	Fondation Congolaise pour la Recherche Médicale - Congo	1	0.2%
**68.**	Global Partnership Development Rep- Africa- ECFMG|FAIMER - Uganda	1	0.2%
**69.**	Institut Pasteur - France	1	0.2%
**70.**	Kenya Medical Research Institute - Kenya	1	0.2%
**71.**	Louisiana State University - USA	1	0.2%
**72.**	Maputo Private Hospital - Mozambique	1	0.2%
**73.**	Massachusetts General Hospital - USA	1	0.2%
**74.**	National AIDS Council - Maputo - Mozambique	1	0.2%
**75.**	National Health Laboratory - South Africa	1	0.2%
**76.**	Resilient and Responsive Health Systems (RRHS) Project, ICAP - DR Congo	1	0.2%
**77.**	Rwanda Ministry of Health - Rwanda	1	0.2%
**78.**	St. John's Research Institute - India	1	0.2%
**79.**	Standford University - USA	1	0.2%
**80.**	Steve Biko Hospital - South Africa	1	0.2%
**81.**	Universidad Peruana Cayetano Heredia - Peru	1	0.2%
**82.**	Université libre de Bruxelles - Belgium	1	0.2%
**83.**	University Medical Central New Orleands - USA	1	0.2%
**84.**	University of California Global Health Institute - USA	1	0.2%
**85.**	University of California Los Angeles - USA	1	0.2%
**86.**	University of North Carolina - USA	1	0.2%
**87.**	UP - Mozambique	1	0.2%
**88.**	VA Palo Alto Healthcare System - USA	1	0.2%
**89.**	VA San Diego HealthCare System - USA	1	0.2%
**90.**	Vanderbilt University - USA	1	0.2%
	**Total**	***456***	***100.0%***
	**Total articles (From Trainees and Networking)**	**Number of mentions**	**%**
**1.**	UEM - Mozambique	80	13.7%
**2.**	UCSD - USA	50	8.6%
**3.**	Stellenbosch University - South Africa	44	7.5%
**4.**	MIHER - Mozambique	39	6.7%
**5.**	Hospital Central de Maputo - Mozambique	26	4.5%
**6.**	University of KwaZulu Natal - South Africa	22	3.8%
**7.**	University of Kinshasa - DR Congo	15	2.6%
**8.**	Johns Hopkins University - USA	11	1.9%
**9.**	Ahmadu Bello University - Nigeria	10	1.7%
**10.**	UniLúrio - Mozambique	10	1.7%
**11.**	University of Cape Coast - Ghana	10	1.7%
**12.**	University of Ibadan - Nigeria	10	1.7%
**13.**	National Institute of Biomedical Research - DR Congo	9	1.5%
**14.**	University of Nairobi - Kenya	9	1.5%
**15.**	Cape Coast Teaching Hospital - Ghana	8	1.4%
**16.**	University of Pittsburgh - USA	8	1.4%
**17.**	Ahmadu Bello University Teaching Hospital - Nigeria	7	1.2%
**18.**	George Washington University - USA	7	1.2%
**19.**	African Centre for Global Health and Social Transformation - Uganda	6	1.0%
**20.**	INS - Mozambique	6	1.0%
**21.**	Monkole Hospital Center - DR Congo	6	1.0%
**22.**	Institute of Human Virology - Nigeria	5	0.9%
**23.**	Jilin University - China	5	0.9%

**24.**	Kenyatta National Hospital - Kenya	5	0.9%
**25.**	Kilimanjaro Christian Medical University College - Tanzania	5	0.9%
**26.**	Université Notre-Dame du Kasayi - DR Congo	5	0.9%
**27.**	University College London - UK	5	0.9%
**28.**	University of California, San Francisco - USA	5	0.9%
**29.**	Addis Ababa University - Ethiopia	4	0.7%
**30.**	Asokoro District Hospital - Nigeria	4	0.7%
**31.**	IHMT Universidade Nova de Lisboa - Portugal	4	0.7%
**32.**	Komfo Anokye Teaching Hospital - Ghana	4	0.7%
**33.**	Kwame Nkrumah University of Science and Technology - Ghana	4	0.7%
**34.**	MISAU - Mozambique	4	0.7%
**35.**	Université Catholique de Bukavu - DR Congo	4	0.7%
**36.**	Université Evangelique en Afrique - DR Congo	4	0.7%
**37.**	University of Lubumbashi - DR Congo	4	0.7%
**38.**	University of Maryland - USA	4	0.7%
**39.**	University of Pretoria - South Africa	4	0.7%
**40.**	Cliniques Universitaires St-Luc - Belgium	3	0.5%
**41.**	Cytel - Canada	3	0.5%
**42.**	Elizabeth Glaser Pediatric AIDS Foundation - USA	3	0.5%
**43.**	Ghent University - Belgium	3	0.5%
**44.**	Hôpital Provincial Général de Référence de Bukavu - DR Congo	3	0.5%
**45.**	Hospital Central de Quelimane - Mozambique	3	0.5%
**46.**	McMaster University - Canada	3	0.5%
**47.**	Rwanda Biomedical Center - Rwanda	3	0.5%
**48.**	South African Medical Research Council - South Africa	3	0.5%
**49.**	Tygerberg Teaching Hospital - South Africa	3	0.5%
**50.**	Universidade do Porto - Portugal	3	0.5%
**51.**	Université Moderne de Kinkole - DR Congo	3	0.5%
**52.**	University College London Hospitals - UK	3	0.5%
**53.**	University of Malawi - Malawi	3	0.5%
**54.**	Uppsala University - Sweden	3	0.5%
**55.**	World Health Organization Health Emergencies Program COVID-19 Response - DR Congo	3	0.5%
**56.**	Centro de Biotecnologia - Mozambique	2	0.3%
**57.**	Harvard Medical School - USA	2	0.3%
**58.**	Kheth'Impilo AIDS Free Living - South Africa	2	0.3%
**59.**	Mbarara University of Science and Technology - Uganda	2	0.3%
**60.**	NIHR Biomedical Research Centre - UK	2	0.3%
**61.**	Official University of Mbuji-Mayi - DR Congo	2	0.3%
**62.**	Ross University School of Veterinary Medicine - Saint Kitts and Nevis	2	0.3%
**63.**	Tulane University - USA	2	0.3%
**64.**	University of Botswana - Botswana	2	0.3%
**65.**	University of Cape Town - South Africa	2	0.3%
**66.**	University of Rwanda - Rwanda	2	0.3%
**67.**	University of Washington - USA	2	0.3%
**68.**	University of Zambia - Zambia	2	0.3%
**69.**	University of Zimbabwe - Zimbabwe	2	0.3%
**70.**	Western Cape Department of Health - South Africa	2	0.3%
**71.**	Albany Medical Center - USA	1	0.2%
**72.**	CISM - Mozambique	1	0.2%
**73.**	Cliniques Universitaires de Lubumbashi - DR Congo	1	0.2%
**74.**	Entebbe Regional Reference Hospital - Uganda	1	0.2%
**75.**	Federal University of Bahia - Brazil	1	0.2%
**76.**	Fondation Congolaise pour la Recherche Médicale - Congo	1	0.2%
**77.**	Global Partnership Development Rep- Africa- ECFMG|FAIMER - Uganda	1	0.2%
**78.**	Institut Pasteur - France	1	0.2%
**79.**	Kenya Medical Research Institute - Kenya	1	0.2%
**80.**	Louisiana State University - USA	1	0.2%
**81.**	Maputo Private Hospital - Mozambique	1	0.2%
**82.**	Massachusetts General Hospital - USA	1	0.2%
**83.**	Memorial Sloan Kettering Cancer Center - USA	1	0.2%
**84.**	National AIDS Council - Maputo - Mozambique	1	0.2%
**85.**	National Health Laboratory - South Africa	1	0.2%
**86.**	Resilient and Responsive Health Systems (RRHS) Project, ICAP - DR Congo	1	0.2%
**87.**	Rwanda Ministry of Health - Rwanda	1	0.2%
**88.**	South African National Biodiversity Institute – South Africa	1	0.2%
**89.**	St. John's Research Institute - India	1	0.2%
**90.**	Standford University - USA	1	0.2%
**91.**	Steve Biko Hospital - South Africa	1	0.2%
**92.**	Universidad de Granada - Spain	1	0.2%

**93.**	Universidad Peruana Cayetano Heredia - Peru	1	0.2%
**94.**	Université libre de Bruxelles - Belgium	1	0.2%
**95.**	University Medical Central New Orleands - USA	1	0.2%
**96.**	University of California Global Health Institute - USA	1	0.2%
**97.**	University of California Los Angeles - USA	1	0.2%
**98.**	University of Coimbra - Portugal	1	0.2%
**99.**	University of North Carolina - USA	1	0.2%
**100.**	UP - Mozambique	1	0.2%
**101.**	VA Palo Alto Healthcare System - USA	1	0.2%
**102.**	VA San Diego HealthCare System - USA	1	0.2%
**103.**	Vanderbilt University - USA	1	0.2%
	Total	***583***	***100.0%***

E. 2. Countries

	Articles from trainees	Mentions	%	Institutions
**1.**	Mozambique	81	64.3%	9
**2.**	USA	20	15.9%	3
**3.**	South Africa	7	4.8%	3
**4.**	China	5	4.0%	1
**5.**	Portugal	5	4.0%	2
**6.**	Belgium	3	2.4%	1
**7.**	Sweden	3	2.4%	1
**8.**	Saint Kitts and Nevis	2	1.6%	1
**9.**	Spain	1	0.8%	1
	Total	***127***	***100.0%***	***22***
	**Articles from networking**	**Mentions**	**%**	**Institutions**
**1.**	Mozambique	93	20.4%	9
**2.**	USA	86	18.9%	20
**3.**	South Africa	78	17.1%	10
**4.**	DR Congo	60	13.2%	13
**5.**	Nigeria	36	7.9%	5
**6.**	Ghana	26	5.7%	4
**7.**	Kenya	15	3.3%	3
**8.**	Uganda	10	2.2%	4
**9.**	UK	10	2.2%	3
**10.**	Canada	6	1.3%	2
**11.**	Rwanda	6	1.3%	3
**12.**	Tanzania	5	1.1%	1
**13.**	Belgium	4	0.9%	2
**14.**	Ethiopia	4	0.9%	1
**15.**	Malawi	3	0.7%	1
**16.**	Portugal	3	0.7%	1
**17.**	Botswana	2	0.4%	1
**18.**	Zambia	2	0.4%	1
**19.**	Zimbabwe	2	0.4%	1
**20.**	Brazil	1	0.2%	1
**21.**	Congo	1	0.2%	1
**22.**	France	1	0.2%	1
**23.**	India	1	0.2%	1
**24.**	Peru	1	0.2%	1
	Total	***456***	***100.0%***	***90***
	**Total articles (From Trainees and Networking)**	**Mentions**	**%**	**Institutions**
**1.**	Mozambique	174	29.8%	12
**2.**	USA	106	18.2%	22
**3.**	South Africa	85	14.6%	11
**4.**	DR Congo	60	10.3%	13
**5.**	Nigeria	36	6.2%	5
**6.**	Ghana	26	4.5%	4
**7.**	Kenya	15	2.6%	3
**8.**	Uganda	10	1.7%	4
**9.**	UK	10	1.7%	3
**10.**	Portugal	8	1.4%	3
**11.**	Belgium	7	1.2%	3
**12.**	Canada	6	1.0%	2
**13.**	Rwanda	6	1.0%	3
**14.**	China	5	0.9%	1
**15.**	Tanzania	5	0.9%	1
**16.**	Ethiopia	4	0.7%	1
**17.**	Malawi	3	0.5%	1
**18.**	Sweden	3	0.5%	1
**19.**	Botswana	2	0.3%	1
**20.**	Saint Kitts and Nevis	2	0.3%	1
**21.**	Zambia	2	0.3%	1
**22.**	Zimbabwe	2	0.3%	1
**23.**	Brazil	1	0.2%	1
**24.**	Congo	1	0.2%	1
**25.**	France	1	0.2%	1
**26.**	India	1	0.2%	1
**27.**	Peru	1	0.2%	1
**28.**	Spain	1	0.2%	1
	Total	***583***	***100%***	***103***
