# Supplementary material for: Fostering Sustainable Biomedical Research Training in Mozambique: A Spin-Off of the Medical Education Partnership Initiative
Source: Ann Glob Health. 2022 Aug 2;88(1):65. doi: 10.5334/aogh.3684 (PMC9354555; doi:10.5334/aogh.3684)
Supplement: Supplementary File 2. — Description of the participation of trainees, mentors and research administrators in international scientific meetings and courses. [file agh-88-1-3684-s2.pdf]

Supplementary file 2: Description of the participation of trainees, mentors and research administrators in international scientific meetings and courses

A. Trainees and mentors

A1. Conferences and symposiums

Nr	Presenter and title	Scientific event name	Role	Country (Location)	Year
**1**	Noormahomed EV. Aspects of Malaria control and drug resistance in Mozambique. BIT's 6th annual world conference of Microbes, 2016, pp. 333.	BIT's 6th annual world conference of Microbes	Mentor	Republic of Korea (Gyeonggi)	2016
**2**	Noormahomed E. Design and implementation of Master programs in underseved region in Mozambique	Seventh Annual CUGH (Consortium of Universities for global Health)	Mentor	USA (San Francisco)	2016
**3**	Langa I	Eighth European & Developing Countries Clinical Trials Partnership (EDCTP) Forum	Trainee	Zambia (Lusaka)	2016
**4**	Chambal L. Characteristics associated with fluid responsiveness and fluid toxicity demonstrated by passive leg rise maneuver in septic patients in a Mozambican emergency department	UCSD CFAR Research day	Trainee	USA (California)	2016
**5**	Zindoga P. Screening and treatment for latent tuberculosis infection of health workers at Maputo Central Hospital, The National reference hospital of Mozambique	UCSD CFAR Research day	Trainee	USA (California)	2016
**6**	Noormahomed E, Ali M & Ismail M [Title not available]	Eighth Annual CUGH Consortium of Universities for Global Health	Mentor	USA (Washington)	2017
**7**	Ali M. The use of ICTs for training health professionals	AFREHealth 1st Annual Symposium	Senior faculty	Ghana (Accra)	2017
**8**	Cossa, M. Assessment of surgical care provided in national health services hospitals in Mozambique	AFREHealth 1st Annual Symposium	Mentor	Ghana (Accra)	2017
**9**	Noormahomed E, Scooley ROC 8566 The Universidade Eduardo Mondlane And University Of California San Diego Partnership, A Paradigm For Institutional And Human Resources Capacity Building. BMJ Global Health 2019;4:A14.	Ninth Edition of the EDTCP Forum	Mentor	Portugal (Lisbon)	2018
**10**	Mocumbi AO. Strengthening Mozambique's Health Post-Graduate Education & Research Systems Through Sustainable Partnerships.	AFREHealth 2nd Annual Symposium	Mentor	South Africa (Durban)	2018
**11**	Noormahomed, E. Building Health Research Capacity in Mozambique: Lessons learned from MEPI and the way forward.	AFREHealth 2nd Annual Symposium	Mentor	South Africa (Durban)	2018
**12**	Chipako P. Integration of information technology into the education of health sciences: Faculty of Health Sciences, UniLurio.	AFREHealth 2nd Annual Symposium	Researcher	South Africa (Durban)	2018
**13**	Noormahomed E.	Ninth Annual CUGH Consortium of Universities for Global Health	Mentor	USA (New York)	2018
**14**	Chambal L. Epidemiologic study to evaluate artemisinin resistance in hospitalized patients with severe malaria in Maputo, Mozambique.	UCSD CFAR Research day	Trainee	USA (California)	2018
**15**	Nhatave C. Lucas G, Wate I, Sáu Z, Patel S, Noormahomed EV, Schooley RT, Taplitz R (2019). Profile of bacterial infectious disease and antimicrobial choices in an urban hospital at Maputo, Mozambique: a prospective observational study. The Lancet Global Health, Volume 7, Supplement 1. 2019, Page S14.	CUGH 10th annual conference	Trainee	USA (Chicago)	2019
**16**	Noormahomed EV. Pathology Postgraduate Program in Mozambique Training, Research, Clinical and Lab Infrastructure	CUGH 10th annual conference	Mentor	USA (Chicago)	2019
**17**	Noormahomed EV. Establishing Master Programs in a Resource-Limited setting through the Medical Education Partnership Initiative: The University of Lurio Experience	CUGH 10th annual conference	Mentor	USA (Chicago)	2019
**18**	Sidat M. Mozambican health system progress towards achieving of the health sustainable development goals (SDG 3)	AFREHealth 3rd Annual Symposium	Mentor	Nigeria (Lagos)	2019
**19**	Sidat M & Palha C. Reviewing medical schools' contribution to the retention of medical graduates within national health service in Mozambique	AFREHealth 3rd Annual Symposium	Mentor	Nigeria (Lagos)	2019
**20**	Noormahomed E. Enhanced Advanced Biomedical Research Training in Mozambique (EABRTM)	AFREHealth 3rd Annual Symposium	Mentor	Nigeria (Lagos)	2019
**21**	Zavale BL. Closing the Gaps on Medical Health	AFREHealth 3rd Annual Symposium	Trainee	Nigeria (Lagos)	2019

Nr	Presenter and title	Scientific event name	Role	Country (Location)	Year
	professional in low income countries through information and communication technologies. The Mozambique experience				
**22**	Banze L. Co-Infection HIV-Schistosoma spp. And intestinal parasites in patients attending the Boane Health Center in Maputo Province, Mozambique	AFREHealth 3rd Annual Symposium	Trainee	Nigeria (Lagos)	2019
**23**	Manuel L. Medical plants used by traditional healers for the treatment of malaria in the Mogovolas district, northern region of Mozambique	AFREHealth 3rd Annual Symposium	Trainee	Nigeria (Lagos)	2019

A2. D43 network meeting

Presenters	Title	Role	Year	Country (Location)
Comia I, Noormahomed E	The prevalence of leptospirose in rodents from Ilha de Moçambique, Nampula-Mozambique	Trainee (Comia I)	2017	Ethiopia (Addis Ababa)
Professor Noormahomed E	Enhanced Advanced Biomedical Research Training for Mozambique (EABRTM).	PD/PI	2018	South Africa (Durban)
Saide A	An evaluation of the dentistry internship program at Lurio University: lessons learned and way forward.	Trainee	2018	South Africa (Durban)
Chirrime I	Evaluation of artemisinin resistance in hospitalized patients with severe malaria in Maputo, Mozambique.	Trainee & Research Assistant	2018	South Africa (Durban)
Cuambe A	The impact of the Program one Student one family.	Trainee	2018	South Africa (Durban)
Professor Mohsin Sidat	Mozambican health system progress towards achieving of the health sustainable development goals (SDG 3)	Mentor	2019	Nigeria (Lagos)
Professor Mohsin Sidat	Reviewing medical schools' contribution to the retention of medical graduates within national health service in Mozambique	Mentor	2019	Nigeria (Lagos)
Professor Noormahomed E	Enhanced Advanced Biomedical Research Training in Mozambique (EABRTM)	Mentor	2019	Nigeria (Lagos)
ZavaLe BL	Closing the Gaps on Medical Health professional in low income countries through information and communication technologies. The Mozambique experience	Trainee	2019	Nigeria (Lagos)
Banze L	Co-Infection HIV-Schistosoma spp. And intestinal parasites in patients attending the Boane Health Center in Maputo Province, Mozambique	Trainee	2019	Nigeria (Lagos)
Manuel L	Medical plants used by traditional healers for the treatment of malaria in the Mogovolas district, northern region of Mozambique	Trainee	2019	Nigeria (Lagos)
Dobe I	The costs of a stroke care in Maputo	Trainee	2020	Online (Zoom)
Miambo R	[No presentation: Trainee panel]	Trainee	2020	Online (Zoom)
Professor Noormahomed E	Enhanced Advanced Biomedical Research Training in Mozambique, Universidade Eduardo Mondlane	PD/PI	2020	Online (Zoom)

A3. Courses

Nr.	Participant's name	Role	Type of training	Title	Country (Location)	Year	Month
**1**	Amélia Mandane	Senior Faculty, UniLúrio	Course	Research in Health Sciences	Portugal (Porto)	2016	May
**2**	Momade Ali	Senior Faculty, UniLúrio	Course (PhD module)	Educative Innovation	Mozambique (Beira)	2016	May
**3**	Eva Dora da Cruz	PhD student, INS	Pratical training	Rotavirus	South Africa (Johannesburg)	2016	April
**4**	Cesar Faria	Senior Faculty, UniLúrio	Training	Quantitative research methods	Spain (Madrid)	2015	November
**5**	Isac Comia	Junior Faculty UniLúrio	Training	Molecular Biology	Mozambique (Maputo)	2016	November
**6**	Professor Emília Noormahomed	PD/PI	Course	35th European Course in Tropical Epidemiology	Portugal (Lisbon)	2016	August
**7**	Momade Ali,	Senior Faculty, UniLúrio	Workshop	Scientific writing	Mozambique	2017	May

Nr.	Participant's name	Role	Type of training	Title	Country (Location)	Year	Month
	Leonardo Manuel, Ibraimo Chabite				(Quelimane)		
8	Laize Botas	Junior Faculty, UniLúrio	Pratical training	Molecular biology	Mozambique (Maputo)	2017	April
9	Marta Cassocera	PhD student, INS	Course (PhD Module)	Biostatistics/Data Analyses and Health Economy at IHMT-UNL	Portugal (Lisbon)	2017	April
10	Irina de Sousa	Trainee, UEM	Pratical training	Molecular Biology	USA (California)	2019	August
11	Regina Miambo	Trainee, UEM	Pratical training	Phylogenetic analysis	South Africa (KwaZulu-Natal)	2019	Novem ber
12	Noémia Nhancupe	Senior Faculty, UEM	Pratical training	Molecular Biology	USA (California)	2019	August

B. Research Administrators

Nr.	Participant's name	Type of training/activity	Title	Country (Location)	Year	Month
1	Iva Chirrime, Margarida Macie and Leonardo Manuel	Workshop	Workshop on grants policy and management training	South Africa - Johannesburg	2016	May
2	Sérgio Noormahomed, Vanessa Vaila, Iva Chirrime, Margarida Macie	Conference	Society of Research Administrators Annual Conference	USA	2016	October
3	Sérgio Noormahomed	Section meeting	Society of Research Administrators Section Meeting	Iceland - Reykjavik	2017	May
4	Sérgio Noormahomed	Workshop	Society of Research Administrator workshop in Research Leadershio	USA	2017	April
5	Sérgio Noormahomed	Conference	SRAI (Society of Research Administration International) Annual Conference	Canada - Vancouver	2017	October
6	Fameti Taero	Training	Resource Mobilization, Organizational Development (Module IV)	Zambia - Lusaka	2018	May-June
7	Sérgio Noormahomed	Training	Change leadership: The path to collaboration	USA	2018	May
8	Nenita Samuel	Training	USAID & CDC financial management & compliance 2018 & beyond	Maputo	2018	April
9	Nenita Samuel	Training	Training Internal controls under the revised “Green Book”	Maputo	2018	April
10	Nicole Mocumbi	Course	Basics of Research Administration	USA - New York	2018	February
11	Sérgio Noormahomed	Annual meeting	Annual Society of Research Administration 2018	USA - Orlando	2018	October
12	Whety Sarmento and José Braz	Training	Fundamentals of research administration	Maputo By MIHER Sergio Noormahomed	2019	No informati on
13	Sérgio Noormahomed and José Braz	Annual meeting	SRAI Annual Meeting 2019	USA - San Fransisco	2019	October
14	Iva Chirrime, Andrea Come, Nicole Mocumbi	Conference	iSRA Virtual Conference	Online	2020	April
15	Seana Daud, Djalmo, Agnesse Cuambe, Noémia Nhancupe, Irina de Sousa, Janny Matuele, Regina Miambo, Milva Nguenha	Course	Training Course on Management Procedures MIHER's Administrative And Research Administration	Maputo By MIHER Sergio Noormahomed	2020	October
