# Supplementary material for: Fostering Sustainable Biomedical Research Training in Mozambique: A Spin-Off of the Medical Education Partnership Initiative
Source: Ann Glob Health. 2022 Aug 2;88(1):65. doi: 10.5334/aogh.3684 (PMC9354555; doi:10.5334/aogh.3684)
Supplement: Supplementary File 3. — Funding agencies of the 59 grants administered by MIHER from 2015 to 2021. [file agh-88-1-3684-s3.pdf]

Supplementary file 3. Funding agencies of the 59 grants administered by MIHER from 2015 to 2021.

	**Funding Agencies**	**Number of grants**	**% of grants**	**Total Funding**
**1.**	Fogarty International Center – NIH	28	47.6%	$ 13,176,735.00
**2.**	German Federal Ministry of Education and Research (BMBF)	3	5.1%	$ 1,803,141.09
**3.**	World Hearth Federation	4	6.8%	$ 333,700.00
**4.**	Pfizer Inc.	2	3.4%	$ 174,262.00
**5.**	Health Resources and Services Administration (HRSA)	2	3.4%	$ 154,150.00
**6.**	Queens Margaret University	1	1.7%	$ 137,458.00
**7.**	Australian Catholic University	2	3.4%	$ 119,699.00
**8.**	STADEMA Foundation	1	1.7%	$ 84,240.00
**9.**	Partners in Health	4	6.8%	$ 71,358.06
**10.**	Primary Care International	1	1.7%	$ 60,538.00
**11.**	UC Global Health Institute	1	1.7%	$ 50,000.00
**12.**	U.S. Civilian Research & Development Foundation	1	1.7%	$ 50,000.00
**13.**	Chain of HOPE	2	3.4%	$ 49,998.00
**14.**	Specialist in Global Health	3	5.1%	$ 47,147.27
**15.**	MBIO Diagnostics, Inc.	1	1.7%	$ 20,000.00
**16.**	Brunel University	1	1.7%	$ 11,250.00
**17.**	Heart Iniative Momemtum Research	1	1.7%	$ 10,000.00
**18.**	Afrisight	1	1.7%	$ 9,980.00
	**Total**	59	100.00%	$ 16,363,656.42
